# Higher medical costs for CKD patients with a rapid decline in eGFR: A cohort study from the Japanese general population

**DOI:** 10.1371/journal.pone.0216432

**Published:** 2019-05-17

**Authors:** Kei Nagai, Chiho Iseki, Kunitoshi Iseki, Masahide Kondo, Koichi Asahi, Chie Saito, Ryoya Tsunoda, Reiko Okubo, Kunihiro Yamagata

**Affiliations:** 1 Department of Nephrology, Faculty of Medicine, University of Tsukuba, Tsukuba, Ibaraki, Japan; 2 Okinawa Heart and Renal Association (OHRA), Naha, Okinawa, Japan; 3 Department of Health Care Policy and Health Economics, Faculty of Medicine, University of Tsukuba, Tsukuba, Ibaraki, Japan; 4 Division of Nephrology and Hypertension, Iwate Medical University, Morioka, Iwate, Japan; University of Mississippi Medical Center, UNITED STATES

## Abstract

To investigate how changes in eGFR can affect medical costs, a regional cohort of national health insurance beneficiaries in Japan was developed from a nationwide database system (Kokuho database, KDB), and non-individualized data were obtained. From 105,661 people, subjects on chronic dialysis and subjects without consecutive medical checkups were excluded. Finally, medical costs in the follow-up year categorized by annual changes in eGFR between baseline and the next year were longitudinally examined in 70,627 people ranging in age from 40 to 74 years. Global mean costs for subjects with a rapid decrease in eGFR (≤-30%/year) were the highest among all ΔeGFR categories. In men, the cost was 1.42 times that for a stable eGFR. A total of 6,268 (19.4%) men and 5,381 (14.0%) women with eGFR <60 ml/min/1.73 m^2^ were identified in the baseline year. The mean cost was higher with a low eGFR than without a low eGFR, and there were also higher proportions newly initiating dialysis in 2014 (low eGFR with rapid decrease in eGFR vs. with stable eGFR: 9.61% vs. 0.02% in women, P<0.001). Moreover, the costs for low eGFR subjects with a rapid decrease in eGFR were more than twice those of non-low eGFR subjects with a rapid decrease in eGFR and also compared to low eGFR subjects with a stable eGFR. Moreover, initiating chronic dialysis was considered one of the major causes of high medical costs in women with rapid eGFR decline. To the best of our knowledge, this is the first study of renal disease using a cohort developed from the KDB system recently established in Japan.

## Introduction

Chronic kidney disease (CKD) is a risk factor for not only progression to end-stage kidney disease (ESKD), but also for the development of cardiovascular disease (CVD) and all-cause mortality [1–7]. Therefore, CKD patients often develop ESKD and CVD, which generally require expensive treatment modalities and hospitalization. Recent cross-sectional studies demonstrated medical cost increases as estimated glomerular filtration rate (eGFR) decreases [8,9]. However, these investigations did not take variations of renal function and proteinuria into consideration. Recently, an annual decline of GFR has attracted attention as a risk factor for mortality, the incidence of ESKD, and the incidence of CVD [10–14], and change in the eGFR is becoming a promising surrogate marker for clinical endpoints [10–17]. Thus, we hypothesized that the costs for CKD patients with a rapid decline in eGFR are much greater than for those with stable renal function and without proteinuria, because of initiating and taking chronic dialysis, intensive medication, or other multidisciplinary treatment of complications along with CKD progression.

To address this issue, using a national health insurance cohort of (Kokumin-Kenkou Hoken in Japanese; in short, Kokuho) beneficiaries in Japan, aged from 40 to 74 years, total medical costs in the follow-up year categorized by annual changes in eGFR between baseline and the next year were longitudinally examined. Furthermore, whether the annual change in eGFR can discriminate medical costs in the subpopulation with CKD determined by reduced GFR and proteinuria at baseline was examined. To the best of our knowledge, the precise relationship between changes in eGFR and medical costs has not been examined. In addition, this is the first attempt to use a large-sized cohort of more than 100,000 people from the recently established Kokuho database (KDB) for research on renal diseases in Japan. These analyses might demonstrate the impact of a rapid decline in renal function in CKD patients on medical costs for treating CKD, initiating chronic dialysis, and other diseases associated with CKD.

## Methods

### Study population

Everyone living in Japan is required to enroll in one of the many insurance systems. Kokuho, which is for self-employed individuals, as well as for retirees and their dependents, covered 40.1% of the total Japanese population in 2012 [18]. The study cohort consisted of Japanese Kokuho beneficiaries, aged 40–74 years, living in Japan. The study population included 105,661 people who had undergone annual specific health checkups in 2012 according to “The Specific Health Check and Guidance in Japan” [19]. Thus, most of the study participants were relatively healthy, community-dwelling residents. Annual claim files from Kokuho in 2014 after the primary survey were linked to the baseline data in the KDB system. The names, address, and any other personalized data of the participants were completely deleted from the linked data to protect their privacy. Moreover, the researchers could only report aggregate data, not individualized data.

**Fig 1:**
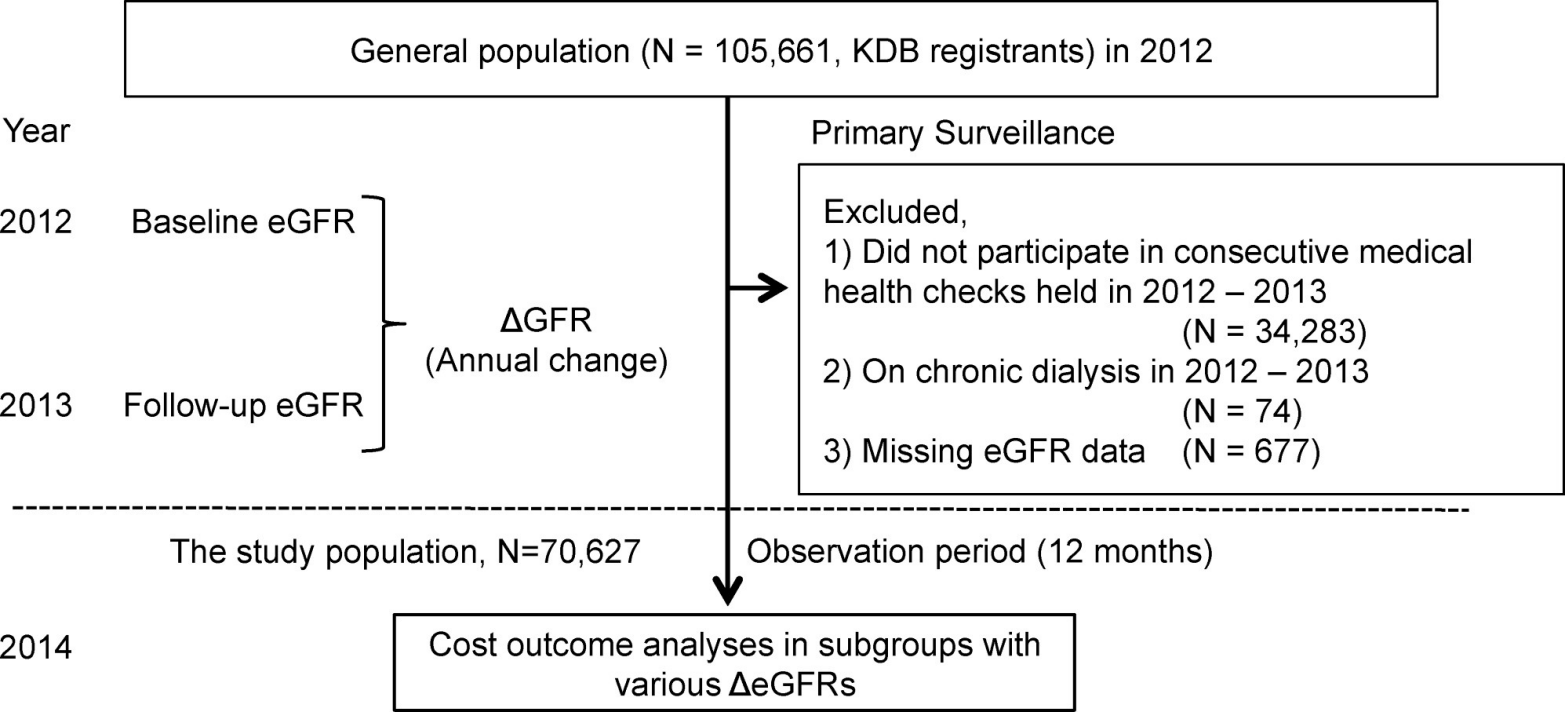
Study design. Strategy for recruitment of the study population, follow-up, and outcome analyses. Abbreviations: KDB, Kokuho database; eGFR, estimated glomerular filtration rate; ΔGFR, annual change in eGFR.

Participants on chronic dialysis (N = 74) until the end of 2013 and not having consecutive medical checkups in 2013 (N = 34,283) and subjects with missing data (N = 677) were excluded. Finally, the subjects included 70,627 people [54.2% were women] from 40 to 74 years of age, for whom all of the data necessary for this study were available, namely, information about age, sex, height, weight, systolic blood pressure, diastolic blood pressure, habitual smoking, use of antihypertensive drugs, lipid-lowering drugs, and hypoglycemic drugs, obtained via a self-reported questionnaire, in addition to data concerning the serum creatinine level and dipstick urine test for proteinuria (**Fig 1**). The institutional review board for ethical issues of the University of Tsukuba approved this study (No. 999, UMIN: 000019774)

### Baseline examination

Serum creatinine was measured using the enzymatic method, and eGFR (ml/min/1.73 m^2^) was calculated using the formula of the Japanese Society of Nephrology [20]. Annual change in eGFR was determined using data in 2012 and 2013, defined as [(eGFR in 2013 –eGFR in 2012) / eGFR in 2012 x (interval months/12)]. As other methodologies were described in detail previously [10], urinalysis by the dipstick method was performed on a single spot urine specimen. Urine dipstick results were interpreted by the medical staff at each local medical institution and recorded as (−), (+/−), (+), (2+), and (3+) as described previously [21]. In Japan, the Japanese Committee for Clinical Laboratory Standards (http://jccls.org/) proposes that all urine dipstick results of (+) should correspond to a urinary protein level of 30 mg/dl. Proteinuria was defined as (+) or more. Blood samples were collected and assayed within 24 hours with an automatic clinical chemical analyzer.

### Follow-up and outcome analyses

The incidence of starting chronic dialysis in this study was defined as no record during the primary survey years (namely, in 2012 and 2013) and an existing record for chronic dialysis therapy in the follow-up year (namely, in 2014). In Japan, since one can discriminate claims for medical costs for hospitalized patients and outpatients, “inpatient” was defined as an individual having a claim as a hospitalized patient of more than one yen, while “outpatient” was defined as having any claim as an outpatient, but no claims as a hospitalized patient. “No claim” was defined as having claims neither as an inpatient nor or as an outpatient during follow-up. Medical costs for the study participants were calculated by summing their claims for the 12 months of 2014.

### Statistical analysis

The outcomes for the analysis were the incidence of starting dialysis during the follow-up period and the mean medical cost among the subpopulations divided by changes in eGFR during the primary survey years. Categorical variables are presented as percentages and continuous variables as means and standard deviation (**Table 1**). Differences in every variable and mean medical cost were first compared among four categories by change in eGFR (≤-30%, -30 to -15%, -15 to +15%, and >+15% per year) using the chi-squared test or one-way analysis of variance (ANOVA), as appropriate. Student’s *t*-test or Mann-Whitney’s test was used as appropriate to comparing mean or median medical costs between stable eGFR (-15 to +15% per year) and other categories of eGFR change. A p value of <0.05 was considered significant. Statistical analyses and graphical analyses were performed using SPSS version 24, Stata version 14 and GraphPad Prism version 6.

**Table 1:** Demographics of the study population.

		Men				
Annual change in eGFR	(%/year)	≤-30	-30 - -15	-15 - +15	>+15	
Study size	(persons)	776	3725	25321	2476	
Age	(years)	62 ± 9	63 ± 9	62 ± 9	62 ± 9	0.056
Systolic blood pressure	(mmHg)	132 ± 17	130 ± 16	128 ± 16	128 ± 16	< 0.001
Diastolic blood pressure	(mmHg)	77 ± 11	77 ± 10	77 ± 10	77 ± 11	0.613
Use of anti-hypertensive drugs	(%)	45.4	42.3	38.8	42.4	0.007
Hemoglobin A1c	(%)	5.9 ± 1.2	5.8 ± 0.9	5.7 ± 0.7	5.8 ± 0.7	< 0.001
Use of hypoglycemic drugs	(%)	13.7	10.4	8.8	9.9	0.032
Triglycerides	(mg/dl)	146 ± 111	142 ± 114	136 ± 104	143 ± 110	< 0.001
Low-density lipoprotein	(mg/dl)	119.1 ± 32.9	118.5 ± 30.1	119.6 ± 29.3	120.1 ± 31.6	0.146
High-density lipoprotein	(mg/dl)	55.7 ± 15.4	55.4 ± 14.5	55.7 ± 14.2	56.3 ± 14.8	0.098
Use of lipid-lowering drugs	(%)	17.1	15.9	14.8	15.8	0.193
Smoking	(%)	26.5	23.0	19.8	24.3	< 0.001
Body mass index	(kg/m^2^)	25.1 ± 3.6	24.8 ± 3.4	24.8 ± 3.3	24.9 ± 3.5	0.003
Proteinuria at baseline	(+ or more, %)	10.9	8.6	7.4	10.3	0.005
Baseline estimated GFR	(ml/min/1.73 m^2^)	96.6 ± 37.7	85.4 ± 20.7	72.8 ± 15.3	67.5 ± 15.1	< 0.001
Estimated GFR in the next year	(ml/min/1.73 m^2^)	67.2 ± 22.5	69.0 ± 16.5	71.4 ± 14.9	82.1 ± 19.0	< 0.001
		Women				
Annual change in eGFR	(%/year)	≤-30	-30 - -15	-15 - +15	>+15	
Study size	(persons)	1412	5388	28217	3312	
Age	(years)	62 ± 9	63 ± 8	63 ± 8	63 ± 8	0.112
Systolic blood pressure	(mmHg)	128 ± 18	127 ± 17	125 ± 17	125 ± 17	< 0.001
Diastolic blood pressure	(mmHg)	74 ± 10	74 ± 10	74 ± 10	74 ± 11	0.073
Use of anti-hypertensive drugs	(%)	35.1	36.1	32.2	34.2	0.007
Hemoglobin A1c	(%)	5.8 ± 0.9	5.7 ± 0.7	5.7 ± 0.6	5.7 ± 0.7	< 0.001
Use of hypoglycemic drugs	(%)	7.4	6.9	5.4	5.8	< 0.001
Triglycerides	(mg/dl)	120 ± 78	113 ± 70	109 ± 64	113 ± 65	< 0.001
Low-density lipoprotein	(mg/dl)	127.0 ± 31.8	125.3 ± 29.7	127.0 ± 29.9	128.4 ± 32.0	< 0.001
High-density lipoprotein	(mg/dl)	60.7 ± 14.0	61.9 ± 14.5	63.3 ± 14.6	63.5 ± 15.1	< 0.001
Use of lipid-lowering drugs	(%)	22.8	23.5	22.1	22.0	0.067
Smoking	(%)	6.8	5.2	4.3	6.0	0.006
Body mass index	(kg/m^2^)	24.3 ± 4.1	24.1 ± 4.0	23.8 ± 3.8	24.1 ± 4.0	< 0.001
Proteinuria at baseline	(+ or more, %)	4.8	4.2	3.7	5.6	0.071
Baseline estimated GFR	(ml/min/1.73 m^2^)	104.6 ± 32.4	88.7 ± 21.0	73.3 ± 15.2	68.3 ± 14.5	< 0.001
Estimated GFR in the next year	(ml/min/1.73 m^2^)	69.5 ± 17.1	71.0 ± 15.4	72.2 ± 14.9	84.1 ± 19.7	< 0.001

Abbreviations: GFR, glomerular filtration rate; eGFR, estimated glomerular filtration rate

## Results

**Fig 2:**
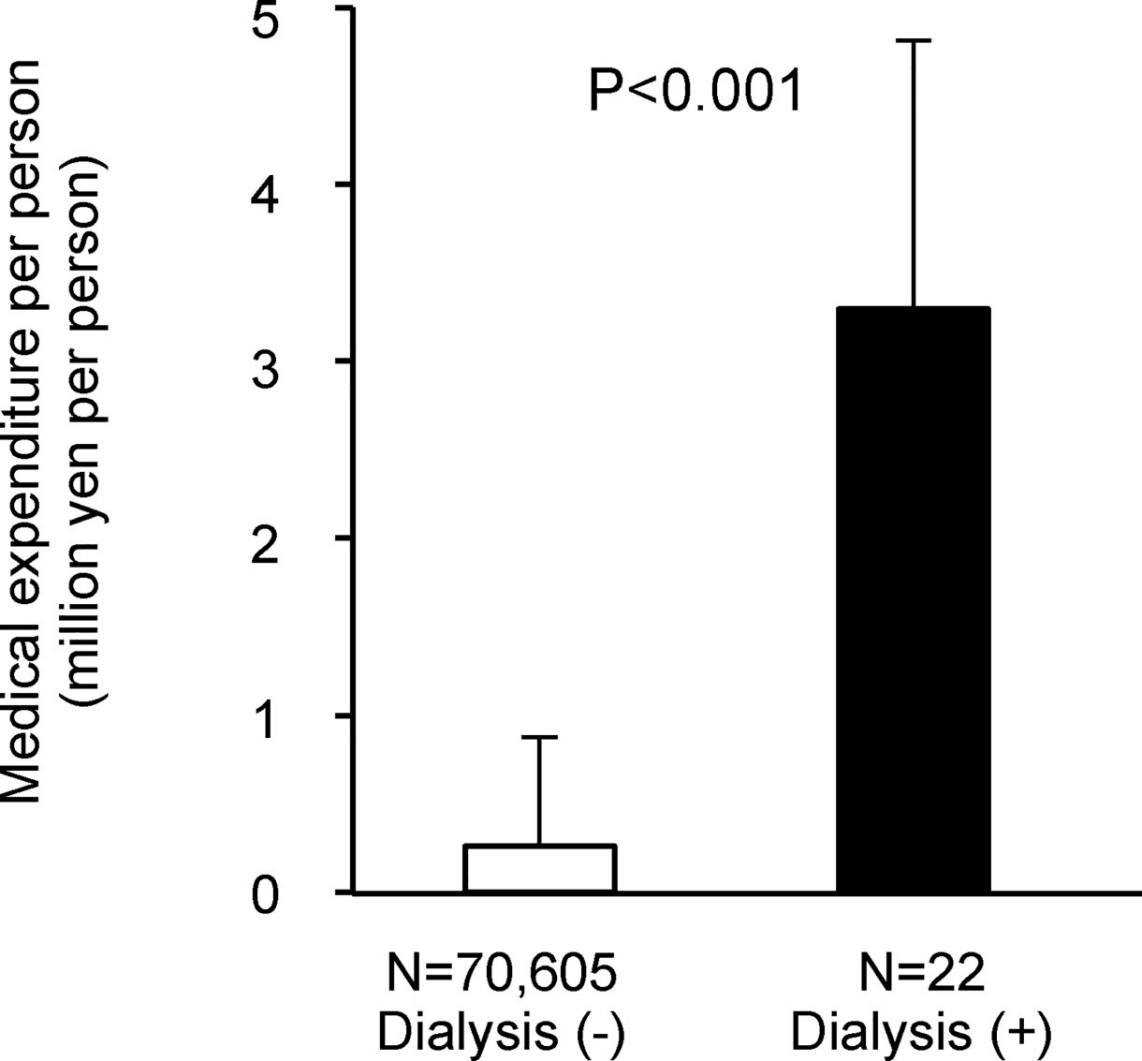
Mean medical costs in the 12 months among the subpopulations starting and not starting chronic dialysis. Patients on chronic dialysis in the baseline year and/or the next year were excluded from the analysis. Therefore, initiation of dialysis occurred in 2014. Error bars show the standard deviations. Abbreviations: eGFR, estimated glomerular filtration rate; ΔGFR, annual changes in eGFR.

During the follow-up period (i.e. 2014), 22 (15 men and 7 women) of the 70,627 study subjects started chronic dialysis (**Figs 1 and 2 and S1 Table**). Their mean medical costs in 2014 are shown in **Fig 2,** and the costs of subjects starting dialysis in 2014 were more than 12 times those who did not start dialysis (3,296,095 yen vs. 261,626 yen).

Comparisons of baseline characteristics among the subpopulations with a rapid decrease (≤ -30% per year), decrease (-30 to -15% per year), stable (-15 to +15% per year), and increase (<+15% per year) in eGFR are shown for men and women separately in **Table 1**. The population with a stable eGFR was the largest (78.4% of men and 73.6% of women), and their eGFR decreased from baseline (i.e. 2012) by 1.4 ml/min/1.73 m^2^ in men and 1.1 ml/min/1.73 m^2^ in women in the next year (i.e. 2013). A rapid decrease in eGFR was seen in 2.4% of men and 3.6% of women, and they showed higher systolic blood pressure, hemoglobin A1c, rate of using antihypertensive drugs and hypoglycemic drugs, triglycerides, rate of habitual smoking, and body mass index, and they tended to have a higher rate of proteinuria.

**Table 2 and S2 Table** show the number of subjects without any claims (# No claim), with claims not including hospitalization (# Outpatient), and with claims including hospitalization (# Inpatient) in 2014 and their proportion in each ΔeGFR category and the mean medical cost per person. The global mean costs for the subjects with a rapid decrease in eGFR were the highest among all ΔeGFR categories, both in men and in women. In men, the cost was 1.42 times that for stable eGFR (385,956 yen vs. 270,897 yen). Particularly in men, but not in women, the proportion of inpatients tended to be higher (15.2%) compared to other ΔeGFR subpopulations (9 to 11%, P = 0.062).

**Table 2:** Mean medical cost, numbers of subjects with or without claims and starting chronic dialysis in the subpopulations based on annual changes in eGFR.

ΔeGFR category	≤-30	-30 - -15	-15 - +15	>+15	ANOVA, *P*	Chi-squared, P
*Men*, *mean cost*, *yen*	385,956	277,329	270,897	323,333	< 0.001	0.009
# No claim	118	508	3600	313		
%	15.2	13.6	14.2	12.6		0.979
Mean cost, yen	0	0	0	0	> 0.999	
# Outpatient	540	2854	19115	1879		
%	69.6	76.6	75.5	75.9		0.029
Mean cost, yen	206,201	189,114	173,416	193,545	< 0.001	
# Inpatient	118	363	2606	284		
%	15.2	9.7	10.3	11.5		0.062
Mean cost, yen	1,594,520	1,359,009	1,360,143	1,538,383	0.141	
# Initiation Dialysis	4	2	7	2		
*Women*, *mean cost*, *yen*	271,159	253,812	244,757	270,362	0.027	0.985
# No claim	153	597	2969	370		
%	10.8	11.1	10.5	11.2		0.999
Mean cost, yen	0	0	0	0	>0.999	
# Outpatient	1145	4384	22931	2660		
%	81.1	81.4	81.3	80.3		>0.999
Mean cost, yen	200,871	187,261	174,125	190,196	< 0.001	
# Inpatient	114	407	2317	282		
%	8.1	7.6	8.2	8.5		0.999
Mean cost, yen	1,341,053	1,342,967	1,257,424	1,381,264	0.414	
# Initiation Dialysis	5	1	1	0		

Abbreviations: eGFR, estimated glomerular filtration rate; ΔGFR, annual changes in eGFR.

When subdividing the subjects by renal function (60 ml/min/1.73 m^2^) for eGFR (G stage 3a or worse) or the result of dipstick proteinuria (1+ or more) in the baseline year (namely, in 2012) according to the criteria of the CKD definition, 6,268 (19.4%) men and 5,381 (14.0%) women with eGFR < 60 ml/min/1.73 m^2^ and 2,530 (7.8%) men and 1,529 (4.0%) women with positive proteinuria were identified (**Table 3 and S3 Table**). Generally, the mean cost for the subjects with a low eGFR or with positive proteinuria was higher than for subjects without a low eGFR. There were also higher proportions of starting dialysis in 2014 (eGFR ≥60 vs. <60; 0.015% vs. 0.031% in men [P<0.001] and 0.000% vs. 0.018% [P<0.001] in women, **Table 3**). In men and in women, mean medical costs for subjects with a rapid decrease in eGFR were obviously the highest among all ΔeGFR categories in cases both with and without renal dysfunction. Moreover, the costs of low eGFR subjects with a rapid decrease in eGFR were more than twice those of non-low eGFR subjects with a rapid decrease in eGFR and also compared to low eGFR subjects with a stable eGFR. Subdividing the subjects with proteinuria in the baseline year also significantly highlighted the massive cost for positive proteinuria subjects with a rapid decrease in eGFR, suggesting that the effect of subdividing by proteinuria is consistent with that of subdividing by eGFR of 60 ml/min/1.73 m^2^ (**Table 3**).

**Table 3:** Mean medical costs, numbers of subjects, and starting chronic dialysis in the subpopulations based on eGFR or proteinuria at baseline and on annual changes in eGFR.

ΔeGFR category		≤-30	-30 - -15	-15 - +15	>+15	ANOVA, P
Low eGFR (<60 ml/min/1.73 m^2^)	*Men*, *%*	9.41	8.99	19.91	33.04	< 0.001
No	Mean cost, yen	349,035	263,326	254,611	295,365	< 0.001
	#	703	3390	20279	1658	
	Incident dialysis, (#)	1	0	3	1	
Yes	Mean cost, yen	741,513	419,036	336,402	380,021	< 0.001
	#	73	335	5042	818	
	Incident dialysis, (#)	3	2	4	1	
	*Women*, *%*	3.68	4.25	15.12	25.18	< 0.001
No	Mean cost, yen	257,686	243,969	234,476	264,007	< 0.001
	#	1360	5159	23951	2478	
	Incident dialysis, (#)	0	0	0	0	
Yes	Mean cost, yen	623,547	475,570	302,478	289,243	< 0.001
	#	52	229	4266	834	
	Incident dialysis, (#)	5	1	1	0	
Proteinuria at baseline	*Men*, *%*	10.89	8.60	7.43	10.25	0.011
Negative	Mean cost, yen	356,600	262,744	262,257	304,439	< 0.001
	#	687	3390	23348	2215	
	Incident dialysis, (#)	1	1	6	1	
Positive	Mean cost, yen	641,752	425,617	366,523	477,199	< 0.001
	#	84	319	1874	253	
	Incident dialysis, (#)	3	1	1	1	
Missing consecutive urinalysis	#	5	16	99	8	
	*Women*, *%*	4.75	4.25	3.74	5.55	0.127
Negative	Mean cost, yen	257,793	246,704	240,559	264,323	< 0.001
	#	1,343	5138	27049	3114	
	Incident dialysis, (#)	0	0	0	0	
Positive	Mean cost, yen	546,868	416,280	337,436	380,172	< 0.001
	#	67	228	1051	183	
	Incident dialysis, (#)	5	1	1	0	
Missing consecutive urinalysis	#	2	22	117	15	

Abbreviations: eGFR, estimated glomerular filtration rate; ΔGFR, annual changes in eGFR.

**Fig 3:**
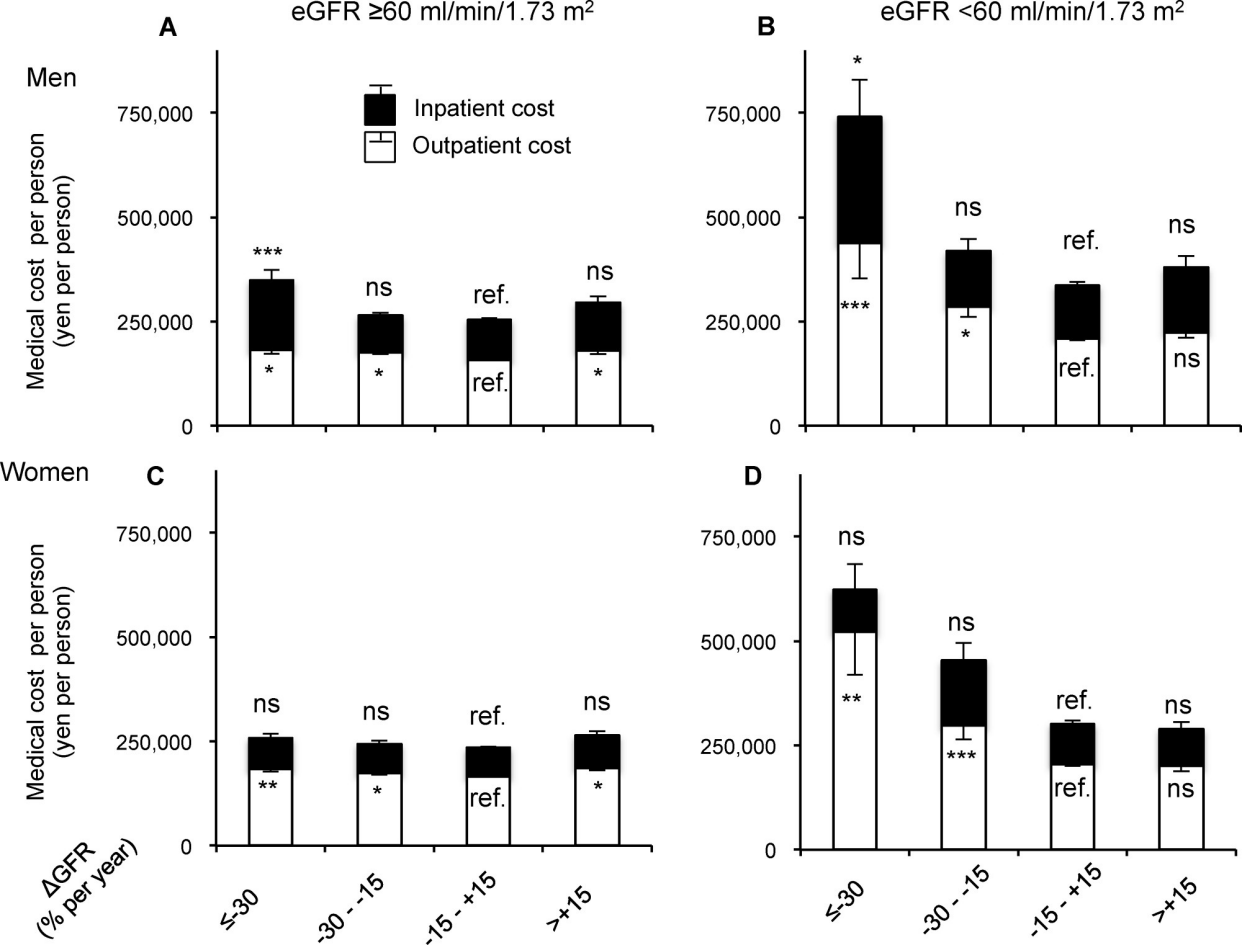
Mean inpatient costs and outpatient costs in the subpopulations based on eGFR at baseline and on annual changes in eGFR. Mean inpatient cost (black bars) and mean outpatient cost (white bars) with standard error of the mean (error bars) shown by category of ΔGFR. The costs for subjects with preserved renal function at baseline (eGFR ≥60 ml/min/1.73 m^2^) have significant but small differences between a rapid decrease in eGFR (-30 to -15% per year) and stable eGFR (-15 to +15% per year) as the reference in men (**A**) and women (**B**). Subjects with reduced renal function at baseline (eGFR <60 ml/min/1.73 m^2^) have dramatically higher costs when their eGFR rapidly decreases compared to the stable (reference) group in men (**C**) and women (**D**). *P<0.05, **P<0.01, ***P<0.001 when comparing to the reference (-15 to +15% per year). Abbreviations: eGFR, estimated glomerular filtration rate; ΔGFR, annual changes in eGFR; ref., Reference.

To demonstrate the impact of hospitalization on medical costs of each ΔeGFR subpopulation, inpatient costs and outpatient costs were further analyzed separately and compared using the mean cost for subjects with a stable eGFR as the reference (**Fig 3 and S4 Table**). In subjects with eGFR ≥60 ml/min/1.73 m^2^, the mean costs for inpatients and for outpatients were significantly, but slightly, higher in men with a rapid decrease in eGFR than in the reference group (**Fig 3A**). In men with eGFR <60 ml/min, the mean cost for inpatients was more than double that compared to the reference group (127,548 yen vs. 304,259 yen), as well as for outpatients (208,855 yen vs. 437,254 yen, **Fig 3B**). In women with eGFR <60 ml/min, specifically outpatients, the mean cost was 2.5 times higher than the reference (204,816 yen vs. 521,292 yen, **Fig 3D**).

## Discussion

This study examined how the changes in renal function affect medical costs in the following year using the database of the KDB system to identify subsequent results of health checkups and medical claims. As far as we know, there have been no previous attempts to use cohort data from the recently established KDB system for research on renal diseases. The present strategy that linked three years (2012 to 2014) succeeded in identifying the subpopulation that started chronic dialysis with eGFR data in the prior two years (**Fig 1**). Throughout Japan, the number of patients starting chronic dialysis was 39,344 in 2016 [22], and the crude rate compared to the general population (127 million) was estimated to be 0.031%. This rate is almost the same as found in this study population, 22 of 70,627 (0.031%), though the age of the subjects ranged from 40 to 75 years, which is the age for annual specific health checkups.

CKD is a risk factor for progression to ESKD, development of CVD, and all-cause mortality [1–7]. Therefore, patients with CKD often develop ESKD requiring chronic dialysis and life-threating complications such as CVD requiring intensive care and hospitalization. As a result, it was demonstrated that medical costs increase as eGFR decreases [8,9]. However, that evidence was based on only one test during screening of serum creatinine and proteinuria. Recently, change in eGFR has been considered to predict the incidence of ESKD and its complications including CVD, and it could be used as a promising surrogate marker for clinical outcomes [15–17], but there has been little evidence on whether changes in eGFR are involved in actual medical costs as consequences of ESKD and other causes.

Definitions and the cutoff point of ΔeGFR have not been settled in clinical studies. However, ΔeGFR was defined as [(eGFR at baseline–eGFR at last follow-up) / eGFR at baseline] in most studies, and a 30% decline in eGFR during follow-up that ranged from one to three years was used for the cut-off based on predicting ESKD [23,24]. They recommended using repeated creatinine measurements with a 2- or 3- year interval rather than a one-year interval, because a longer-term observation period results in more accurate evaluation of ΔeGFR and better prediction of the incidence of ESKD [17,23,24]. The present data used an interval of “approximately” one-year, that is no more than two years. Therefore, to pursue analytical accuracy, we consider it is better to correct by the interval of measurement, because it was 12.6 ± 2.4 (mean ± standard deviation) months in men and 12.6 ± 2.5 months in women.

Subjects with a rapid decrease in eGFR generally have worse co-morbid conditions (hypertension, hyperglycemia, and dyslipidemia) and worse physical status (higher BMI) compared to other ΔeGFR categories (**Table 1**). As a consequence, it was thought that they would incur the highest gross mean cost in the following year (**Table 2**). The major component of the higher burden in men seems to be the low outpatient proportion and the high inpatient proportion, suggesting a higher frequency of diseases requiring hospitalization in men with a rapid decrease in eGFR. Complications in progressive CKD patients, generally speaking CVD, though there was no evidence in this study, are considered to be the major determinants of medical costs in the men of this study population. On the other hand, in women, a rapid decrease in eGFR had little effect on mean medical costs compared to a stable eGFR (271,159 yen vs. 244,757 yen, **Table 2**), partially because of no difference in the frequency of inpatient and outpatient visits among ΔeGFR categories, unlike in men.

Decreases in eGFR in CKD and non-CKD patients should be assessed for the risk of incident and all-cause mortality, because the eGFR slope in CKD with eGFR <60 ml/min/1.73 m^2^ is more significant and sensitive than eGFR ≥60 ml/min/1.73 m^2^ [25]. Consistent with this, subdividing the study population by eGFR or proteinuria can highlight the importance of ΔeGFR in women for medical costs and starting chronic dialysis (**Table 3**). Women with CKD and a rapid decrease in eGFR have a much higher frequency of starting chronic dialysis compared to those with a stable eGFR with CKD (9.61% vs. 0.02% in women [P<0.001, Chi-squared test], **Table 3**). Therefore, starting and being on chronic dialysis appear to be the major causes of the higher mean cost in women with CKD and a rapid decrease in eGFR.

To the best of our knowledge, this is the first study to examine medical costs in a large-sized (over a 100,000 subjects) general population with consecutive results for proteinuria and renal function, consecutive medical claims, and dependency on chronic dialysis. Therefore, the present strategy had the strength to perform sub-analyses of men and women enrolled and to identify the frequency of hospitalizations for calculating inpatient costs and outpatient costs separately. However, this study also has several limitations. First, the purpose of the medical costs was not completely identified. Second, measurement of biochemical parameters including proteinuria and serum creatinine to determine subcategories was performed only once per year. Third, this methodology cannot identify each participant because of the research design that obtained reports composed of non-individual data; therefore, it is not possible to know the risk of disease incidence and hospitalization with adjustment for co-morbid conditions. Finally, this method cannot discriminate which missing claim means death or moving during 2014.

## Conclusion

The results of the present study showed that the costs for CKD patients with a rapid annual decline of eGFR were twice those of patients with stable CKD in the following year. Moreover, starting chronic dialysis appeared to be one of the major causes of medical costs in rapid eGFR decliners in the women of this study population. To the best of our knowledge, this is the first study of renal disease using a large-sized cohort developed from the recently established KDB system in Japan.

## Supporting information

S1 Table Supplemental data for Fig 2. (XLSX)

S2 Table Supplemental data for Table 2. (XLSX)

S3 Table Supplemental data for Table 3. (XLSX)

S4 Table Supplemental data for Fig 3. (XLSX)
